# The use of the GIS Kriging technique to determine the spatial changes of natural radionuclide concentrations in soil and forest cover

**DOI:** 10.1186/s40201-014-0130-6

**Published:** 2014-10-25

**Authors:** Turgay Dindaroğlu

**Affiliations:** Faculty of Forestry, Department of Forest Engineering, Kahramanmaras Sutcu imam University, 46100 Kahramanmaras, Turkey

**Keywords:** Natural radionuclide, Kriging technique, Soil, Forest cover

## Abstract

**Background:**

The distribution of radionuclides occurring naturally in the earth depends on bedrock characteristics. Therefore, the spatial distribution of radionuclides is not uniform. Consequently, radionuclide information is vitally important in determining and monitoring the spatial variation of the radionuclide concentrations that are over the limits for the sustainable environment and human health.

**Methods:**

This research was carried out using GIS methods and geostatistical analysis as Kriging techniques to reveal the spatial variation of the 226Ra, 232Th and 40 K concentrations of natural radionuclides in the Çoruh and Aras Basin. The spatial variations of the detected radionuclides were correlated with soil groups and forest cover.

**Results:**

In the study area, 43.17% of the concentration of 40 K, 26.67% of the concentration of 226Ra and 28.16% of the 232Th concentration was determined to be over the average limits. Concentrations of radionuclides that are over the average limits have been found to be on basalt and chestnut soils. Brown and reddish brown soils have a low concentration of the spatial distribution of the radionuclides. Statistically positive correlations were detected (0.865 **) between the 226Ra and 232Th. In addition, a positive relationship between forest cover and 226Ra and a negative relationship between 232Th and 40 K were identified.

**Conclusions:**

Excessive exposure to radiation may cause cancer and hereditary diseases. Ecological environments include the soil and the plants. Hence, the periodical monitoring of the spatial variation in concentrations of radionuclides is very important for the health of future generations.

## Background

Radioactive features have existed in our world since its formation. High concentrations of natural radionuclides are found in volcanic, phosphate, granite and salt rock. Rain and other water discharge crumble these rocks into very small pieces and mixes them into the groundwater. Thus, rocks increase the natural radioactivity of the soil.

The direction of the movement and the speed of the radionuclides in the soil depends on natural processes (e.g., soil structure, content of the plant species, irrigation conditions, weather conditions and accumulation) [1,2]. In some areas, the natural radionuclide concentrations are above the established limits according to UNSCEAR. If the concentration of natural radioactive elements goes over the average limits, there can be negative effects on human health [3]. Therefore, the spatial distribution of the concentrations of natural radionuclides in the soil has to be determined. It is possible to use CBS and geostatistical analysis to obtain these values.

Geostatistical techniques are a useful component of GIS applications that are frequently applied. Geostatistics involves the analysis and estimation techniques used to obtain the value of a variable dispersed in time and location.

Kriging is one of the best and most widely-known techniques used in spatial linear predictions. Kriging methods have different flexible forms, according to the survey area and data [4–7]. Kriging can also reveal the reliability of the estimated surface [8,9].

Geostatistical methods also allow for examining a relationship with spatial variations of radionuclides between forest cover and soil groups. Radioactive elements in the soil do not indicate a uniform distribution in the earth. Therefore, the concentrations of radionuclides should be checked regularly as an important step in protection from the negative effects of radioactivity [10–12].

Radionuclide concentrations may be caused by the high amount of organic matter in the soil. As such, radionuclides can be absorbed by the forest soil [13,14]. Some radionuclide compounds build up in the humic acids in the soil organic layer [15,16].

Measuring the radioactivity concentration in the soil, as well as the concentrations in the plants and the water, is necessary to estimate the concentrations of radionuclides [17,18]. This study used ^226^Ra, ^232^Th and ^40^ K concentration values measured periodically by TAEK [19]. Spatial distributions were determined according to UNSCEAR (2000) [3], who used a kriging technique in the Çoruh and Aras Basin and the surrounding areas. The statistical relationships between the spatial variations, forest cover and soil groups were analyzed.

## Materials and methods

**Figure 1:**
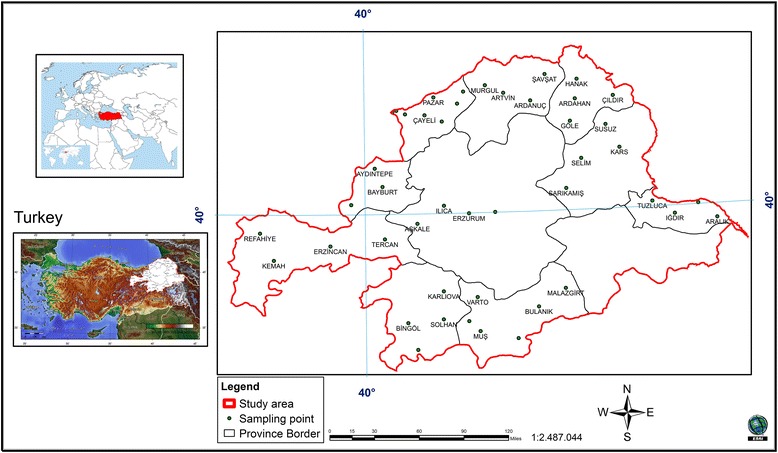
**Location of study area.**

This research was carried out in 10 provinces in eastern Turkey: Rize, Bayburt, Erzurum, Artvin, Ardahan, Kars, Iğdır, Muş, Bingöl and Erzincan. The bounding geographical coordinates of the study area are 39°50´07´´ to 39°46´17´´ north latitudes and 38°26´52´´ to 44°35´46´´ east longitudes (Figure 1). The study area is 9.815.000 ha in size. Forest areas were detected using local Forest Management Plan data [20].

### Sampling methods and analysis

Concentrations of radioactive elements (^226^Ra, ^232^Th and ^40^ K) for determining soil sampling were carried out by the provincial offices of the Ministry of Environment and Urbanization. Surface soil sampling was conducted at 46 points. The concentration of radioactivity in the surface soil samples was determined by the Atomic Research Council of Turkey [19].

### Risk assessment

The average natural radioactivity rate, the concentration range and the average values are presented in Table 1 [3]. To determinate the concentration of the natural radionuclides, soil samples from 10 cm thickness in 1 km^2^ land were collected in the research area.

**Table 1:** **Natural radioactivity the concentration range and average values**

	**Natural radionuclides (Bq/kg)**		
	^**226**^ **Ra**	^**232**^ **Th**	^**40**^ **K**
Concentration range	17-60	11-64	140-850
Average values	35	45	400

### Geostatistical analysis

To conduct the geostatistical analysis, the "Kriging" interpolation technique was used within the spatial analyst extension module in ArcGIS 9.3 software. The spatial analyses were carried out with prepared maps using this technique. Concentrations of ^226^Ra, ^232^Th and ^40^ K were determined for the distribution area. The experimental variogram model was constructed using the Kriging method, with data obtained from the research area. The spatial transformation was performed to determine the most appropriate model to use with the parameters of the generated maps.

The ordinary Kriging formula is as follows [8,21]:

$$ Z\left({\mathrm{S}}_0\right)={\displaystyle \sum_{i=1}^N}{\lambda}_iZ\left({\mathrm{S}}_{\mathrm{i}}\right) $$

where:

Z(s*i*) is the measured value at the location (*i*th),

λ_i_ is the unknown weight for the measured value at the location (*i*th) and

s_0_ is the estimation location.

The unknown weight (λ_p)_ depends on the distance to the location of the prediction and the spatial relationships among the measured values.

The statistical model estimates the unmeasured values using known values. A small difference occurs between the true value Z(s_0_) and the predicted value, ∑λ_i_ Z(s_i)_. Therefore, the statistical prediction is minimized using the following formula:

$$ {\left[Z\left({\mathbf{S}}_0\right)-{\displaystyle \sum_{i=1}^N}{\lambda}_iZ\left({\mathbf{S}}_i\right)\right]}^2 $$

The Kriging interpolation technique is made possible by transferring data into the GIS environment. In this way, analysis in areas that have no data can be conducted. The following criteria were used to evaluate the model: the average error (ME) must be close to 0 and the square root of the estimated error of the mean standardized (RMSS) must be close to 1 [22]. While implementing the models, the anisotropy effect was surveyed.

## Results and discussion

Anisotropic variogram models were preferred. The ^226^Ra, ^232^Th and ^40^ K concentration values showed a directional change. The spatial dependencies (Nugget/Sill ratio) were found to be related to the degree of autocorrelation between the sampling points. If the spatial dependence was higher between the sample points, the spatial correlation was very high. The spatially dependent variables were classified as: strongly spatially dependent if the ratio was ≤25%, mid-spatial-dependent if the ratio was 25% - 75% and weakly spatially dependent if the ration was ≥75% [4,23–26].

Because the spatial dependence was strong, the variables did not differ over short distances. In this research area, spatial dependence was too high for ^232^Th (16.91%). Spatial dependence was identified as normal for ^226^Ra and ^40^ K (25.88% and 70.60%), respectively. An effective spatial dependence distance was found between 254369.7 and 375276 meters (Table 2).

**Table 2:** **Model parameters**

	**Model**	**Regression function**	**Nugget, (C**_**o**_ **)**	**Range, A**	**Sill, (C**_**o**_ **+ C)**	**Nugget/Sill, (%)**	**ME** ^**1**^	**RMSSE** ^**2**^
^226^Ra	Exponential	0.2695 x +18.32	48.35	254369.7	186.83	25.88	0.004	1.06
^232^Th	Exponential	0.5063 x +15.33	50.74	304696.1	300.03	16.91	0.008	0.89
^40^ K	Stable	0.1596 x +381.99	41767.52	375276.0	59158.77	70.60	0.007	0.997

ME^1^: mean standard error.

RMSSE^2^: estimated standardized mean of error of mean square root.

The sample point data will involve converting the spatial data of the ^226^Ra, ^232^Th and ^40^ K concentrations used in the interpolation for kriging. The lowest error rate models were chosen; they were the “*Exponential*” and “*Stable*” models. The maps were produced and field data were obtained in accordance with this kriging model.

### Spatial distribution of ^226^Ra:

The prediction map, according to the optimized model, was determined during the cross-validation process. The ^226^Ra concentration prediction map shows the log ^226^Ra. The dataset for the ^226^Ra concentration has a high kurtosis and is positively skewed, so it is not a normal distribution. The data log transformation was applied to be closer to a normal distribution. After the log transformation was conducted, the ^226^Ra concentrations were found to be approximately normally distributed.

**Figure 2:**
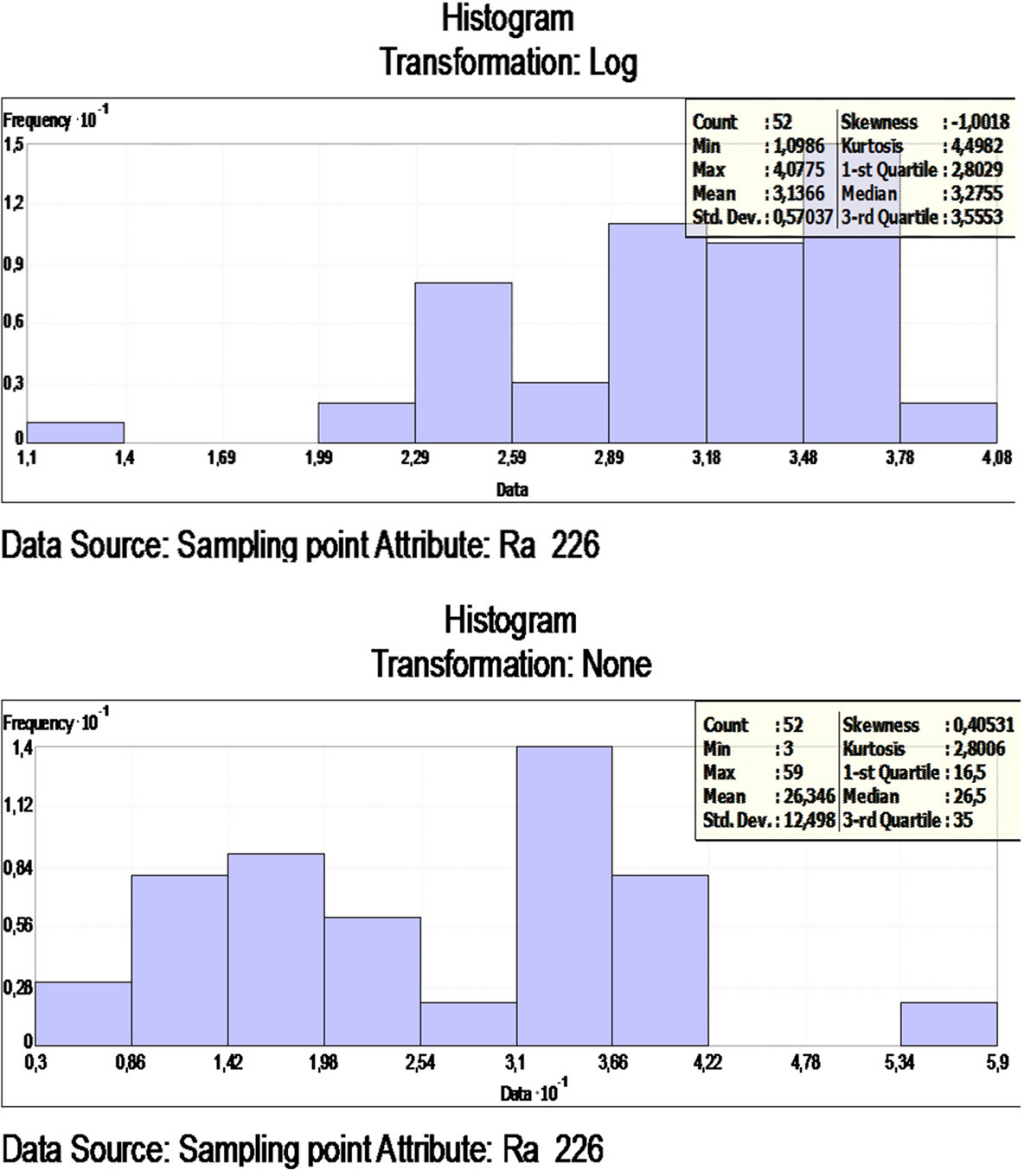
**Frequency distribution of the** ^**226**^ **Ra and Log** ^**226**^ **Ra.**

**Figure 3:**
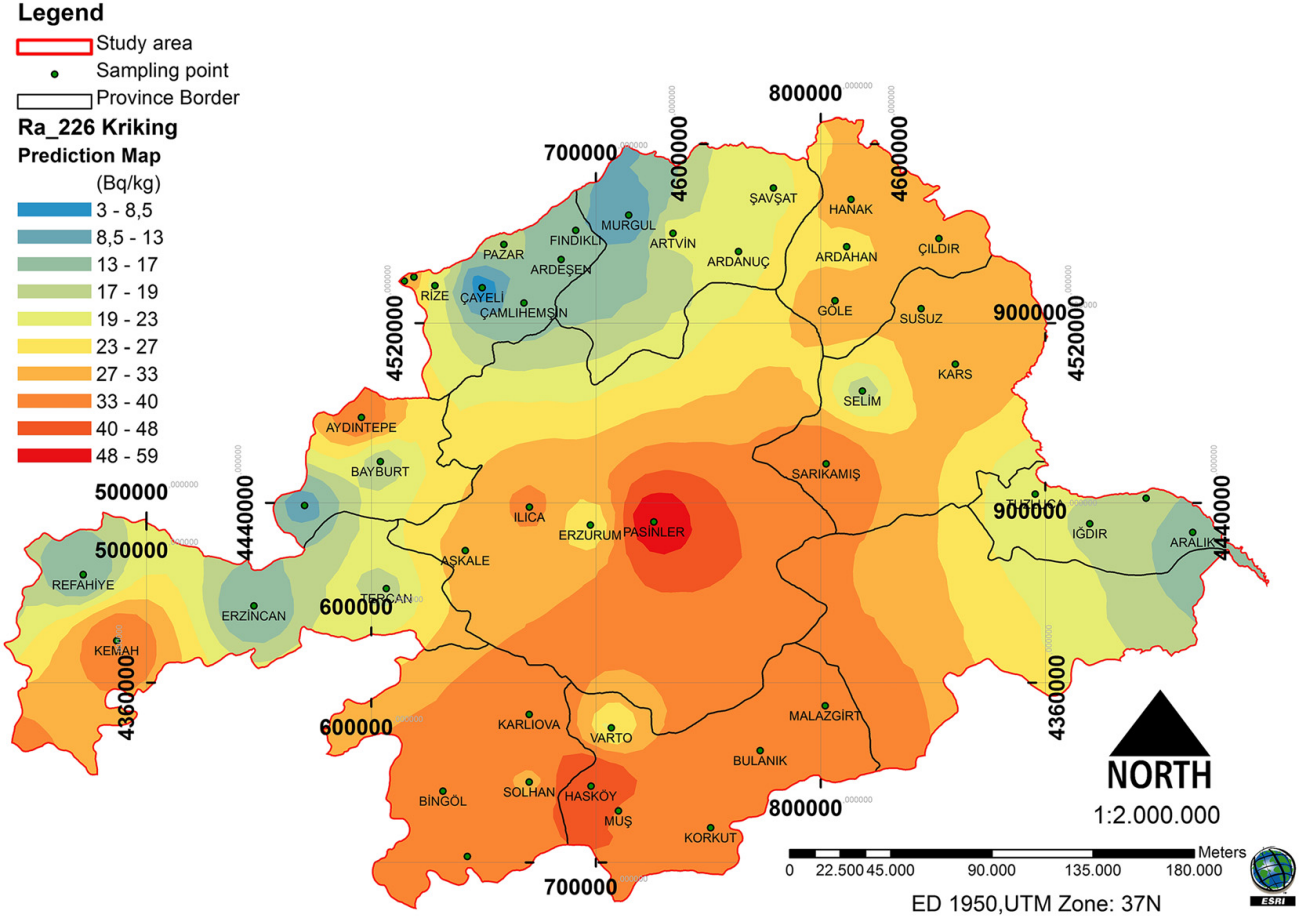
**Spatial prediction map for the ordinary kriging interpolation of** ^**226**^ **Ra.**

The histogram of ^226^Ra and log transformations data is presented in Figure 2. To check the data, the best model was applied to the cross-validation of the spatial correlation of the ^226^Ra concentration of the study area. A comparison of the ME and the RMSSE for the log ^226^Ra illustrates that the exponential model and its parameters were best for the ^226^Ra concentration. The exponential model has the best fit with the nugget effect (C_o_); it is equal to 48.35, a sill (C_o_ + C) equal to 186.83 and a range of influence equal to 254369.7. The ratio of the nugget variance to the sill is expressed in percentages equal to 25.88% (Table 2). This value is greater than 25% and less than 75%. Thus, the ^226^Ra distribution has a moderate spatial dependence in the study area. A spatial prediction and distribution map for the ordinary kriging interpolation of ^226^Ra is presented in Figure 3.

### Spatial distribution of ^232^Th

**Figure 4:**
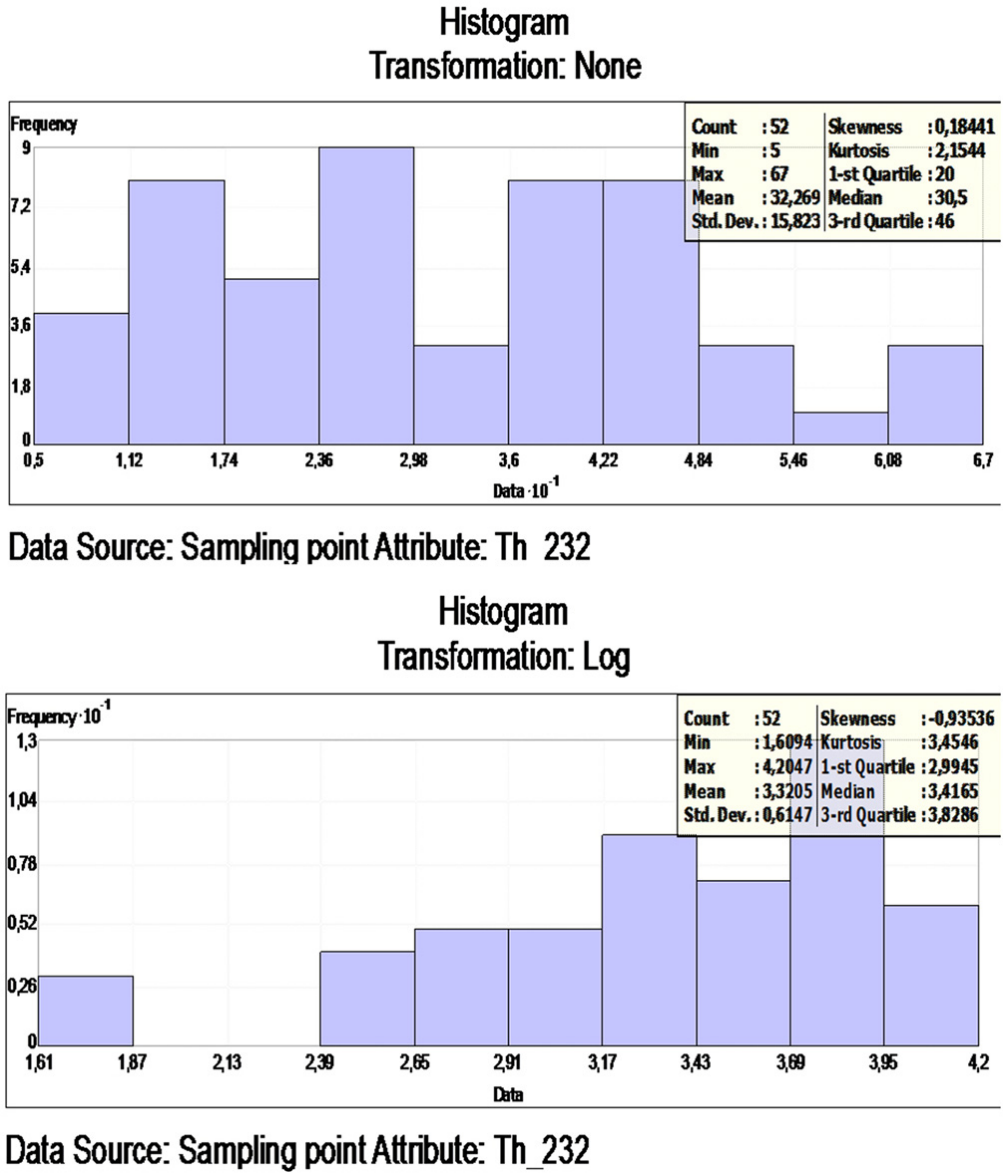
**Frequency distribution of the** ^**232**^ **Th and Log** ^**232**^ **Th.**

**Figure 5:**
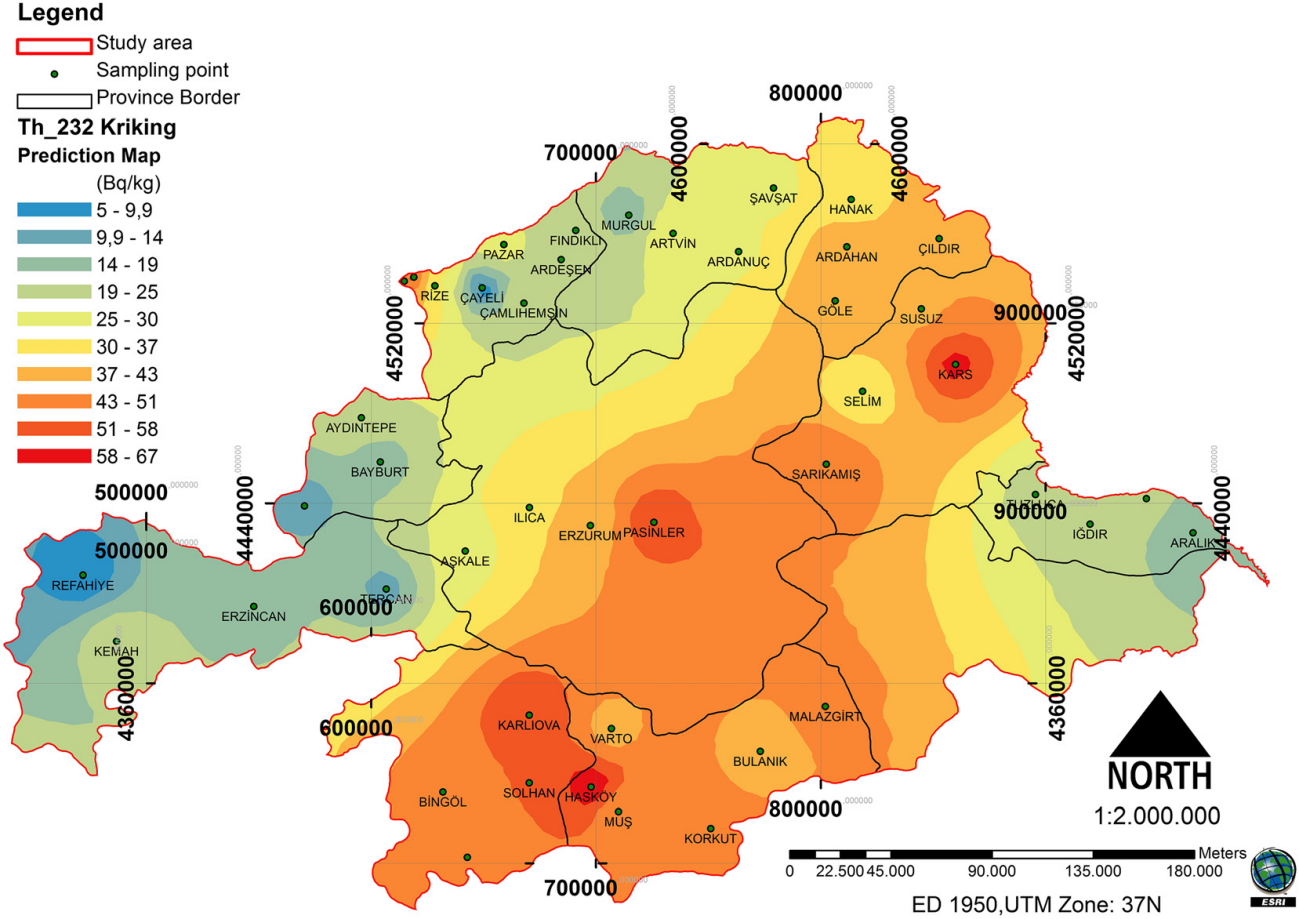
**Spatial prediction map for the ordinary kriging interpolation of** ^**232**^ **Th.**

The dataset of the ^232^Th concentration has a high kurtosis and is positively skewed, so it is not a normal distribution. The data log transformation was applied, so could be closer to a normal distribution. After the log transformation was conducted, the ^232^Th concentrations were approximately normally distributed. The histogram of ^232^Th and log-histogram transformation data is presented in Figure 4. To check that the best model was applied, a cross-validation of the spatial correlation of the ^232^Th concentration of the study area was conducted. A comparison of ME and the RMSSE for log ^232^Th shows that the exponential model and its parameters are the more appropriate for the ^232^Th concentration. The exponential model has the best fit, with the nugget effect (C_o_) being equal to 50.74, a sill (C_o_ + C) equal to 300.03 and a range of influence equal to 304696.1. The ratio of the nugget variance to the sill expressed in percentages is equal to 16.91% (Table 2). This value is smaller than 25; thus, the ^232^Th distribution has a powerful spatial dependence in the study area. A spatial prediction and distribution map for the ordinary kriging interpolation ^232^Th is presented in Figure 5.

### Spatial distribution of ^40^ K

**Figure 6:**
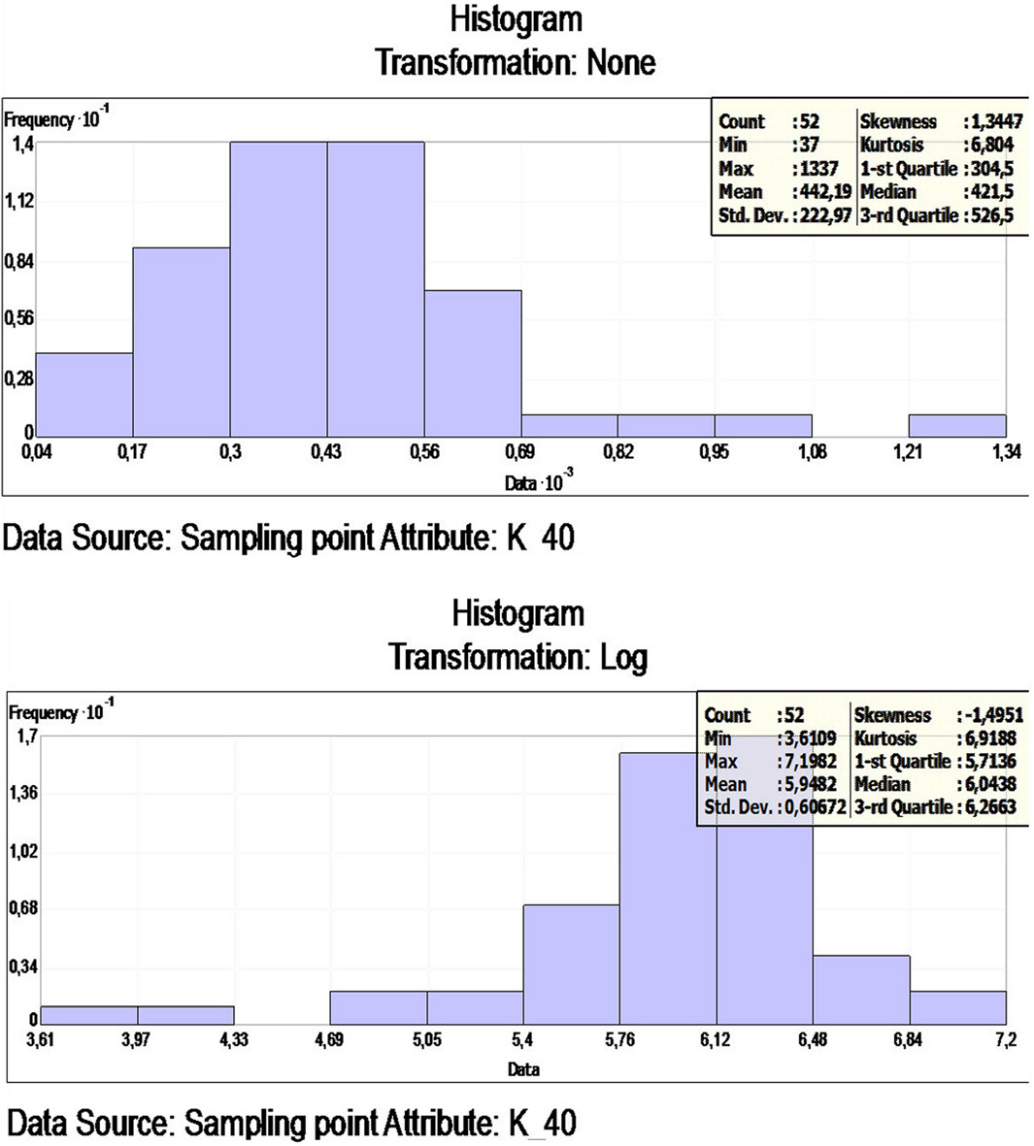
**Frequency distribution of the** ^**40**^ **K and Log** ^**40**^ **K.**

An optimized model determined from the cross-validation process and the ^40^ K concentration prediction map shows the log ^40^ K. The dataset for the ^40^ K concentration has a high kurtosis and is positively skewed, so it is not a normal distribution. The data log transformation was applied, so the data would be closer to a normal distribution. After the log transformation, the ^40^ K concentrations were found to be approximately normally distributed. The histogram of ^40^ K and the log transformation data is presented in Figure 6.

**Figure 7:**
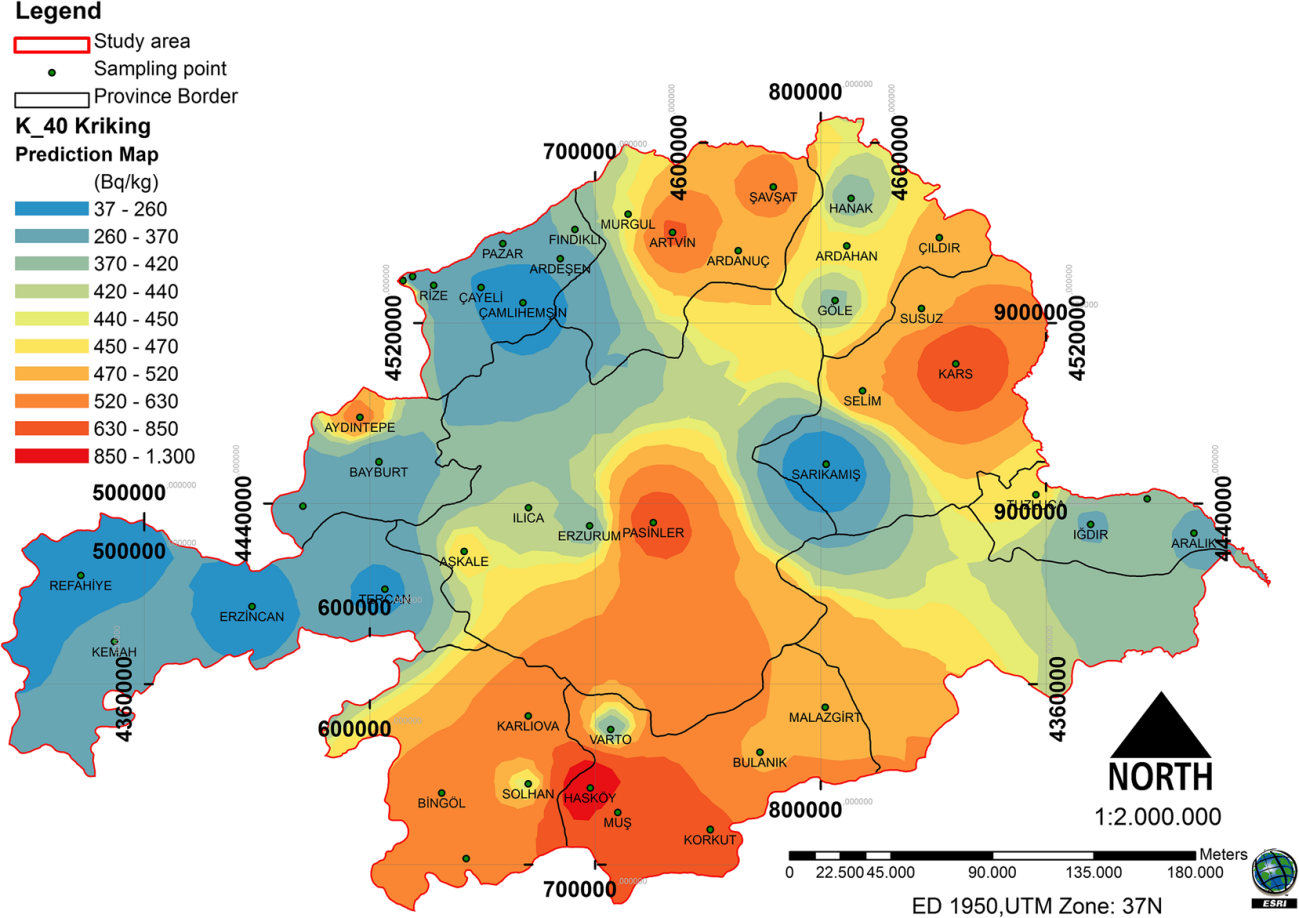
**Spatial prediction map for the ordinary kriging interpolation of** ^**40**^ **K.**

A comparison of the ME and the RMSSE for log ^40^ K shows that the stable model and its parameters is the best model that illustrates for ^40^ K concentration. The stable model has the best fit with the nugget effect (C_o_) being equal to 41767.52, a sill (C_o_ + C) equal to 59158.77 and a range of influence equal to 375276. The ratio of nugget variance to sill is expressed in percentages equal to 70.60% (Table 2). This value is greater than 25 and less than 75%; thus, the ^40^ K distribution has a moderate spatial dependence in the study area. The spatial prediction and distribution map for the ordinary kriging interpolation ^40^ K is presented in Figure 7.

### The relationship between forest cover and natural radionuclides

**Figure 8:**
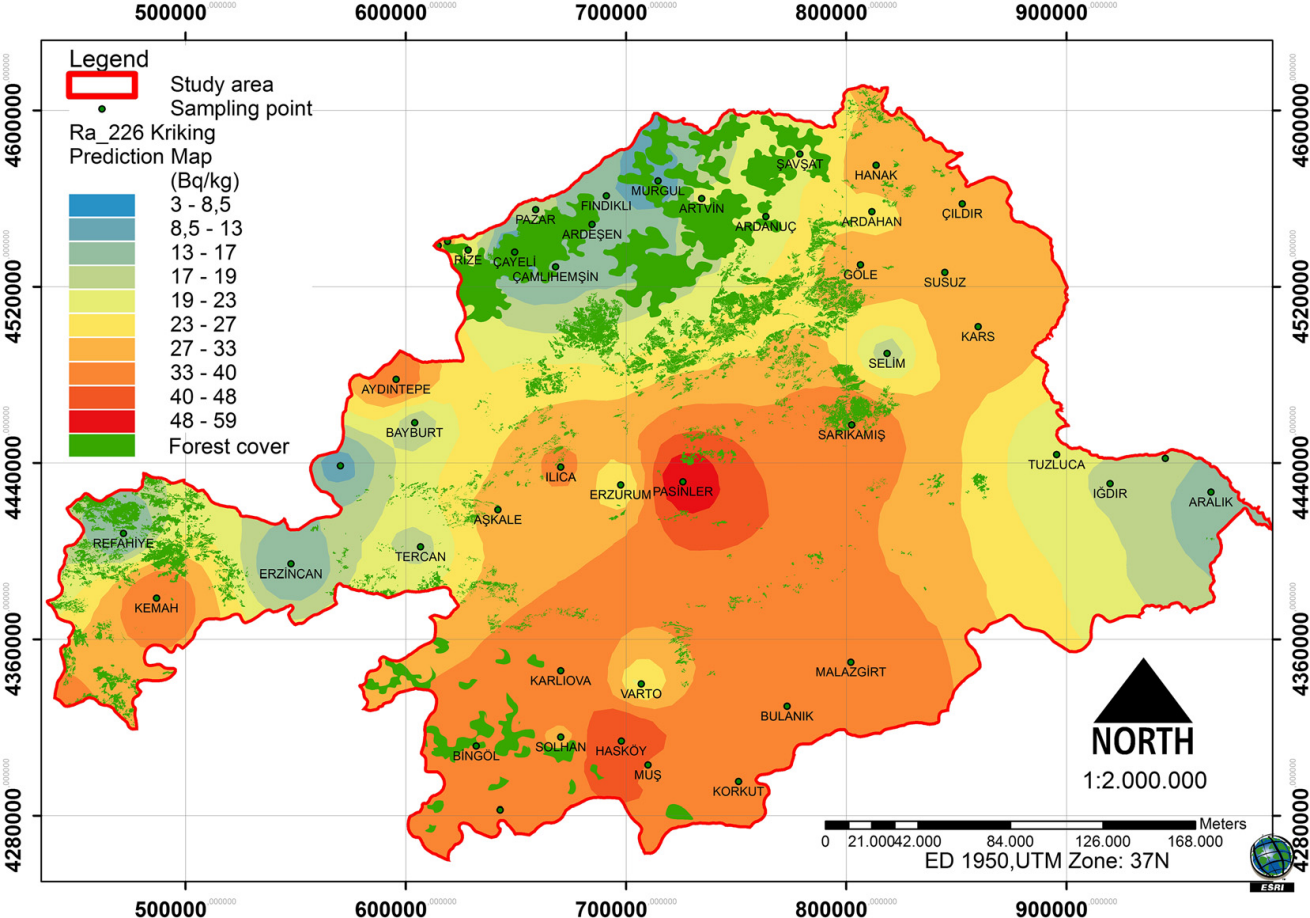
^**226**^ **Ra and spatial distribution of forest cover.**

**Figure 9:**
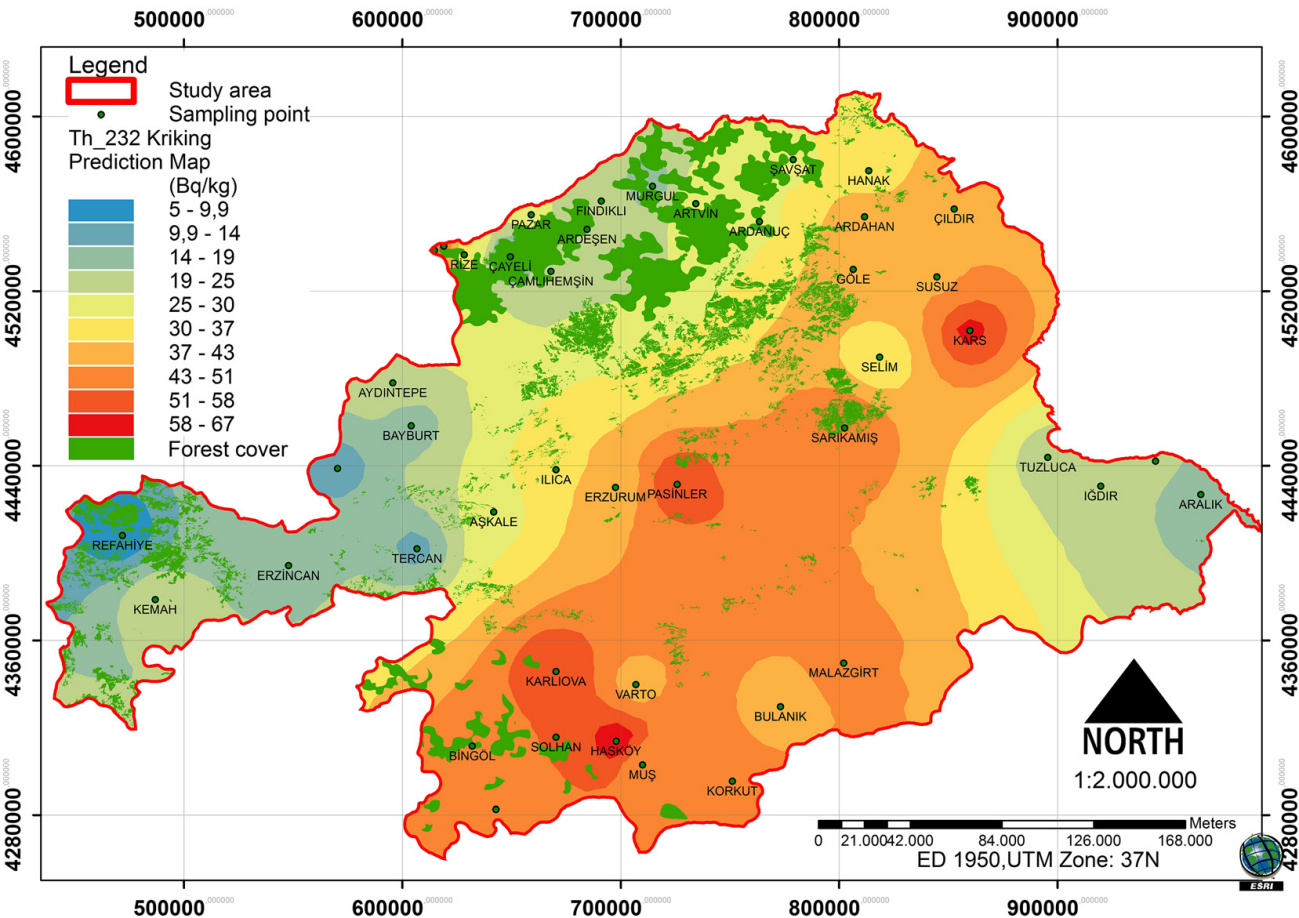
^**232**^ **Th and spatial distribution of forest cover.**

**Figure 10:**
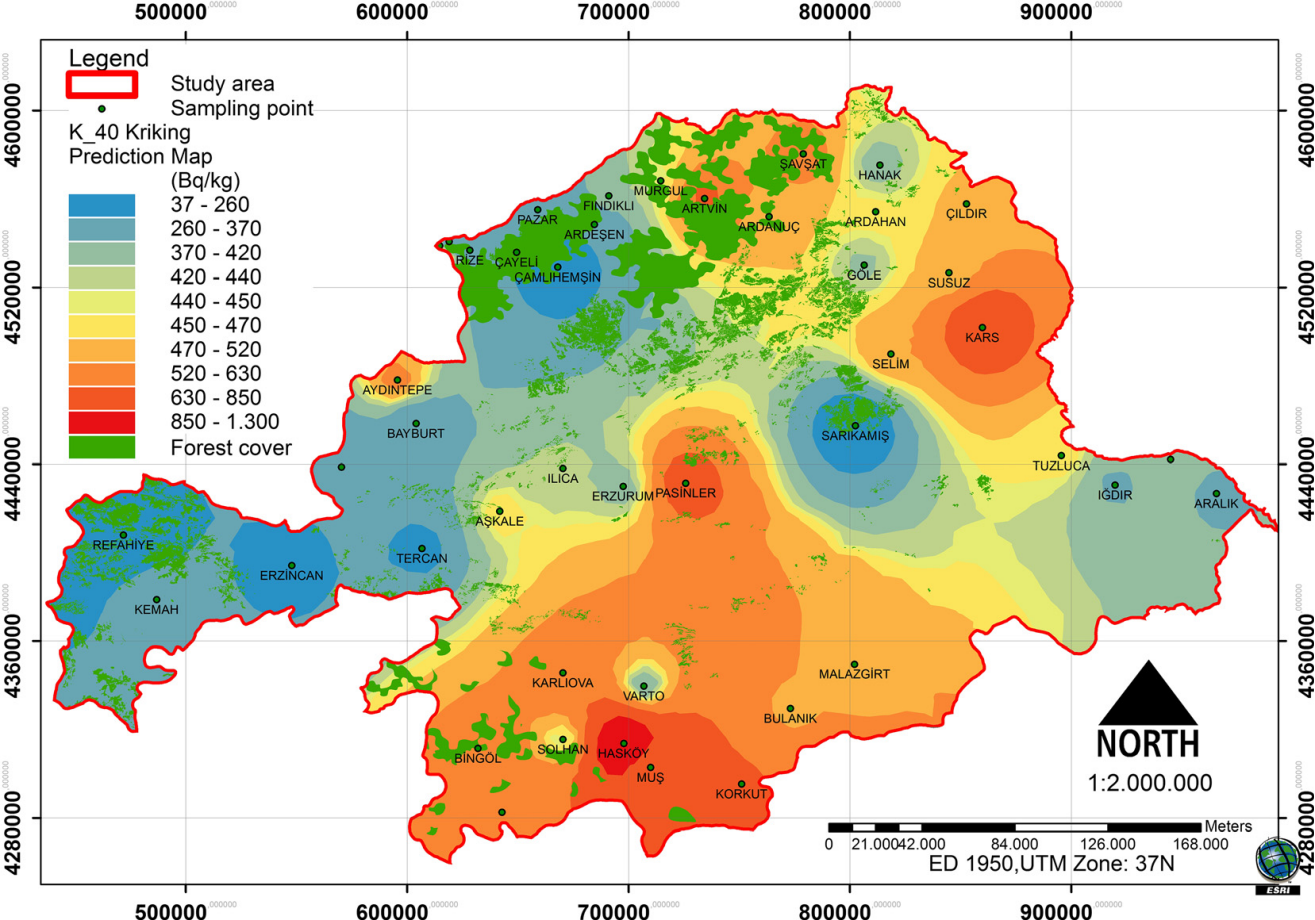
^**40**^ **K and spatial distribution of forest cover.**

In the study area, the spatial distribution was analyzed in relation to the forest ecosystems and the radionuclides (Figures 8, 9 and 10). Between the ^226^Ra and ^232^Th, a positive increase in the 0.01 significance level (0.865**) was detected. Between the ^40^ K and ^232^Th, a positive increase in the 0.05 significance level (0.718*) was detected. Between the forest cover and ^226^Ra, a negative relationship was identified. Between the ^232^Th and ^40^ K, a positive relationship was identified (Table 3). The radionuclide concentrations were found to depend on the soil humus content [27]. Different contents and amounts of organic matter in forest ecosystems effect the spatial variation of radionuclides in the soil.

**Table 3:** **Correlations test result between radionuclides and forest cover**

**Parameters**	**Radionuclides**			**Forest cover**
	^**226**^ **Ra**	^**232**^ **Th**	^**40**^ **K**	
^**226**^ **Ra**	1			
^**232**^ **Th**	0,865^**^	1		
^**40**^ **K**	0,592	0,718^*^	1	
**Forest cover**	−0,008	0,096	0,232	1

**Correlation is significant at the 0.01 level (2-tailed).

*Correlation is significant at the 0.05 level (2-tailed).

### Spatial changes of great soil groups and radionuclides in the soil

**Figure 11:**
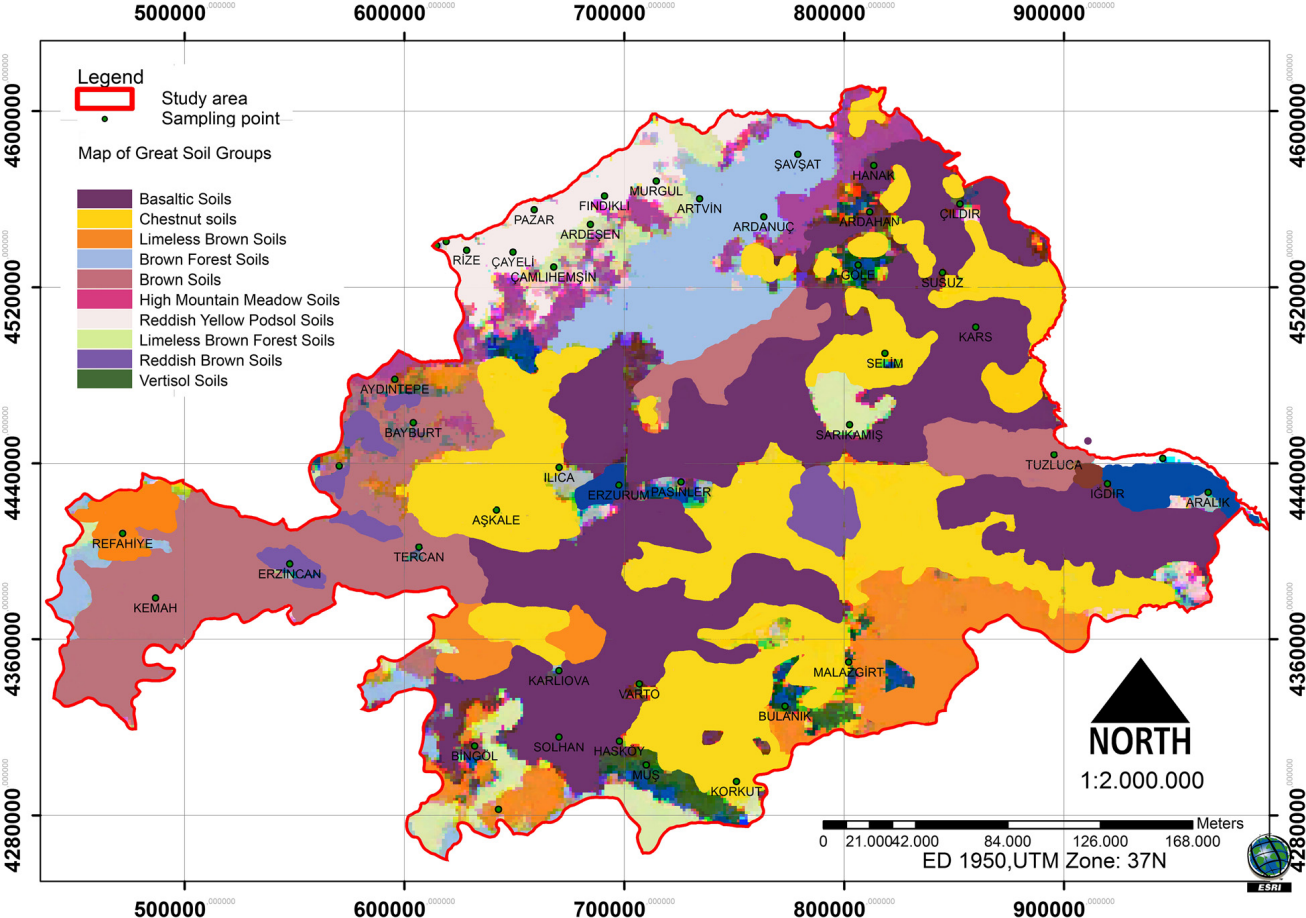
**Map of great soil groups.**

The soil group’s map of the study area is presented in Figure 11. It was determined that 43.17% of the concentration of ^40^ K, 28.16% of the concentration of ^226^Ra and 26.67% of the concentration of ^232^Th was above the average UNSCEAR (2000) concentrations.

Within the study area, 17.49% of the Basaltic soils and 11.51% of the Chestnut soils had above average concentrations of the ^40^ K radionuclides. In addition, 10.8% of the Basaltic soils and 9.59% of the Chestnut soils had above average concentrations of ^226^Ra radionuclides. It was also determined that 10.47% of the Basaltic soils and 9.32% of the Chestnut soils had above average concentrations of ^232^Th radionuclides (Table 4). Consequently, all basalt and chestnut soil field locations had above average radionuclide concentrations.

**Table 4:** **Spatial changes between radionuclides and great soil groups**

	**Spatial changes of the radionuclides concentration (Hectare)**					
**Great soil goups**	^**40**^ **K**		^**226**^ **Ra**		^**232**^ **Th**	
	**Under optimal concentrations (<400 Bg/kg)**	**Upper optimal concentrations (>400 Bg/kg)**	**Under optimal concentrations (<35 Bg/kg)**	**Upper optimal concentrations (>35 Bg/kg)**	**Under optimal concentrations (<45 Bg/kg)**	**Upper optimal concentrations (>45 Bg/kg)**
Bazaltic Soils	1187656	1717242	1915127	989771	1876826	1028072
Chesnut Soils	1101871	1129844	1290559	941156	1316923	914792
Limeless Brownish Soils	428955	582383	620178	391160	762213	249125
Limeless Brown Forest Soils	94789	228346	119664	203471	135446	187689
Vertisol Soils	0	76254	0	76254	0	76254
Brown Forest Soils	464221	287303	684116	67408	684116	67408
Brown Soils	1505651	0	1505651	0	1505651	0
High Mountain Soils	262129	159430	421559	0	421559	0
Reddish Yellow Podsol Soils	438377	56680	495057	0	495057	0
Reddish Brown Soils	94684	0	0	94684	0	94684

Brown soils, High Mountain soils, Reddish Brown soils and Reddish Yellow Podsol soils in areas containing high concentrations of ^226^Ra and ^232^Th radionuclides had no spatial distribution pattern. In areas with Brown soils and Reddish Brown soils, high concentrations of ^40^ K radionuclides had no spatial distribution pattern (Table 4).

In summary, changes in the concentrations of radionuclides in the soil depend on the formation of iron oxide and other elements. Some of the acids formed in the soil, through calcium carbonate found in the environment, prevent the retention of the radionuclides. Therefore, radionuclide concentrations in the rocks can be reduced with calcium carbonate; this, in turn, reduces the level of external radiation [28,29]. According to the Anonymous [30], some rocks and a soil type typical of the specific radioactivity values was identified in the follow: For ^40^ K; Granite (1000 Bq/kg), clay stone (700 Bq/kg), Sandstone (350 Bq/kg) Basalt (250 Bq/kg) and limestone (90 Bq/kg). For ^232^Th; Granite (80 Bq/kg), clay stone (50 Bq/kg), Sandstone (10 Bq/kg), Basalt (10 Bq/kg) and Limestone (7 Bq/kg). Local distribution values can vary greatly according to changing areas [31,32].

## Conclusions

Over time, the infiltration of radionuclides has resulted in high radionuclide concentrations in the lower soil layers. The radionuclides in these lower soil layers can move upwards through the roots of plants and be transferred to the plant during the growth process. Since radionuclides can have detrimental health effects on humans, it is important to determine the spatial variation of concentrations of radioactive elements.

This research was conducted to examine to spatial distribution of natural radioactive element (^226^Ra, ^232^Th and ^40^ K) concentrations and their relationship with soil groups and forest cover using the Kriging method. According to the statistical analyses, positive correlations were detected between the ^226^Ra and the ^232^Th (0.865**), as well as between the ^40^ K and ^232^Th (0.718*). Negative correlations between forest cover and ^226^Ra were found, while positive correlations between ^232^Th and ^40^ K were detected.

The basalt and chestnut soils in the study area were found to have above average concentrations of radionuclides. The Brown soils, High Mountain soils, Reddish Brown soils and Reddish Yellow Podsol soils did not have high concentrations of ^226^Ra and ^232^Th. The Brown soils and the Reddish Brown soils also did not have high concentrations of ^40^ K.

Radionuclides are present in different concentrations in the soil, plants and water, which comprise parts of the basic food chain. Excessive exposure to radiation can lead to cancer; it is also the cause of hereditary diseases. Therefore, spatial variations of radioactive element concentrations need to be monitoring for the sustainability of a healthy environment.

## References

[1] Theodorsson P (1997). Measurements of Weak Radioactivity.

[2] TAEK: **Resource of natural radiation.** http://www.taek.gov.tr/bilgi-kosesi/184-radyasyon-insan-ve-cevre/radyasyonla-birlikte-yasiyoruz/501-dogal-radyasyon-kaynaklari.html Accessed on: 11.03.2014.

[3] UNSCEAR: **Report on sources and effects of ionizing radiation to the general assembly, United Nations, Vienna.** 2000, http://www.unscear.org/unscear/en/publications/2000_1.html Accessed on: 25.02.2014.

[4] Robertson GP (1987). **Geostatistics in ecology**: **interpolation with known variance**. Ecology.

[5] Cressie NAC (1993). Statistics for Spatial Data.

[6] Goovaerts P (1997). Geostatistics for Natural Resources Evaluation.

[7] Ecker MD (2004). Geostatistics: Past, Present and Future, Encyclopedia of Life Support Systems (EOLSS), Developed under the Auspices of the UNESCO.

[8] Isaaks EH, Srivastava RM (1989). An Introduction to Applied Geostatistics.

[9] Brown RB, Huddleston JH (1991). Presentation of statistics data on Map units to the user. In spatial variabilities of soil and landform. SSSA Special.

[10] Khan HM, Khan K, Atta MA, Jan F (1994). Measurement of gamma activity of soil samples of Charsadda district of Pakistan. J Chem Soc Pakistan.

[11] Rühm W, Kammerer L, Hiersche L, Wirth E (1996). Migration of 137Cs and 134Cs in different forest soil layers. J Environ Radioact.

[12] Rosén K, Öborn I, Lönsjö H (1999). Migration of radiocaesium in Swedish soil profiles after the Chernobyl accident, 1987–1995. J Environ Radioact.

[13] Bergman R (1994). The distribution of radioactive caesium in boreal forest ecosystems. In Nordic Radioecology, the Transfer of Radionuclides through Nordic Ecosystems to Man. Studies in Environmental Science.

[14] Gorham E (1963). A comparison of natural and fallout radioactivity in Ontario soils under pine. Can J Bot.

[15] Degering D, Schlenker S, Unterricker S (2000). Radionuclide Behaviour in Natural Organic Matter (Peat, Cola and Forest Soil Surfaces).

[16] Sokolik GA, Ivanova TG, Leinova SL, Ovsiannikova SV, Kimlenko IM (2001). Migration ability of radionuclides in soil-vegetative cover of Belarus after Chernobyl accident. Environ Int.

[17] UNSCEAR (1993). Sources and Effects of Ionizing Radiation, Report of the General Assembly With Scientific Annex B.

[18] Hölgye Z, Malý M (2000). Sources, vertical distribution, and migration rates of ^239, 240^Pu, ^238^Pu, and ^137^Cs in grassland soil in three localities of central Bohemia. J Environ Radioact.

[19] TAEK: Turkey Atomic Energy Agency: *Turkey Atlas of Environmental Radioactivity.* Ankara: 2014. http://www.taek.gov.tr/radyasyon-izleme/turkiye-cevresel-radyasyon-atlasi.html Accessed on: 26.03.2014.

[20] OGM (2012). Forest Cover Map.

[21] ESRİ: **The principels of geostatistical analysis.** 2013, 54. http://maps.unomaha.edu/Peterson/gisII/ESRImanuals/Ch3_Principles.pdf 22.02.2014.

[22] Johnston K, Hoef M, Krivoruchko K, Lucas N (2001). Using ArcGIS Geostatistical Analyst.

[23] Clark I (1979). Practical Geostatistics.

[24] Trangmar BB, Yost RS, Uehara G (1985). Application of geostatic to spatial studies of soil properties. Advances in Argon.

[25] Cambardella CA, Moorman TB, Novak JM, Parkin TB, Karlen DL, Turco RF, Konopka AE (1994). Field-scale variability soil properties in Central Iowa soils. Soil Sci Soc Am J.

[26] Erşahin S (1999). Alluvial soil in a field, some physical and chemical properties of the spatial variability of the determination. SU Journal of the Faculty of Agriculture.

[27] Chelmicki V, Mietelski JW, Macharski P, Swicchowicz J (1993). Natural Factors of Cs-137 Rcdistribution in Soil.

[28] NCRP (1975). National Council on Radiation Protection and Measurements.

[29] Özger AG (2005). District of Ceyhan, Yumurtalık and Pozantı Determination of Natural Radioactivity Levels. M.Sc. Thesis.

[30] Anonymous (1995). Federal Ministry for the Environment, Nature Conservation and Nuclear Safety, environmental radioactivity and radiation exposure, Annual Report, Bonn.

[31] Atakan Y (2008). Natural radioactivity, natural and human created in the radiation doses.

[32] Vukašinović I, Đorđević A, Rajković M, Todorović D, Pavlović V (2010). Distribution of natural radionuclides in anthrosol-type soil. Turk J Agric.

